# Inhibition of the Quorum Sensing System, Elastase Production and Biofilm Formation in *Pseudomonas aeruginosa* by Psammaplin A and Bisaprasin

**DOI:** 10.3390/molecules27051721

**Published:** 2022-03-06

**Authors:** Emmanuel T. Oluwabusola, Nursheena Parveen Katermeran, Wee Han Poh, Teo Min Ben Goh, Lik Tong Tan, Oluwatofunmilayo Diyaolu, Jioji Tabudravu, Rainer Ebel, Scott A. Rice, Marcel Jaspars

**Affiliations:** 1 Department of Pharmacy, DDT College of Medicine, Gaborone P.O. Box 70587, Botswana; 2 Natural Sciences and Science Education, National Institute of Education, Nanyang Technological University, 1 Nanyang Walk, Singapore 637616, Singapore; nursheena92@gmail.com (N.P.K.); bengoh93@yahoo.com.sg (T.M.B.G.); liktong.tan@nie.edu.sg (L.T.T.); 3 Singapore Centre for Environmental Life Sciences Engineering, Singapore 637551, Singapore; whpoh@ntu.edu.sg (W.H.P.); rscott@ntu.edu.sg (S.A.R.); 4 Marine Biodiscovery Centre, Department of Chemistry, University of Aberdeen, Aberdeen AB24 3UE, UK; r01oad17@abdn.ac.uk (O.D.); r.ebel@abdn.ac.uk (R.E.); 5 School of Forensic and Applied Sciences, Faculty of Science and Technology, University of Central Lancashire, Preston PR1 2HE, UK; jtabudravu@uclan.ac.uk; 6 The School of Biological Sciences, Nanyang Technological University, Singapore 639798, Singapore; 7 The iThree Institute, The University of Technology Sydney, Sydney, NSW 2007, Australia

**Keywords:** marine sponge, psammaplin, marine natural products, quorum sensing inhibitor, *Pseudomonas aeruginosa*, inhibitor of biofilm formation, elastase inhibitor

## Abstract

Natural products derived from marine sponges have exhibited bioactivity and, in some cases, serve as potent quorum sensing inhibitory agents that prevent biofilm formation and attenuate virulence factor expression by pathogenic microorganisms. In this study, the inhibitory activity of the psammaplin-type compounds, psammaplin A (**1**) and bisaprasin (**2**), isolated from the marine sponge, *Aplysinella* *rhax*, are evaluated in quorum sensing inhibitory assays based on the *Pseudomonas aeruginosa* PAO1 *lasB-gfp*(ASV) and *rhlA-gfp*(ASV) biosensor strains. The results indicate that psammaplin A (**1**) showed moderate inhibition on *lasB-gfp* expression, but significantly inhibited the QS-gene promoter, *rhlA-gfp*, with IC_50_ values at 14.02 μM and 4.99 μM, respectively. In contrast, bisaprasin (**2**) displayed significant florescence inhibition in both biosensors, PAO1 *lasB-gfp* and *rhlA-gfp*, with IC_50_ values at 3.53 μM and 2.41 μM, respectively. Preliminary analysis suggested the importance of the bromotyrosine and oxime functionalities for QSI activity in these molecules. In addition, psammaplin A and bisaprasin downregulated elastase expression as determined by the standard enzymatic elastase assay, although greater reduction in elastase production was observed with **1** at 50 μM and 100 μM. Furthermore, the study revealed that bisaprasin (**2**) reduced biofilm formation in *P. aeruginosa.*

## 1. Introduction

The discovery of antibiotics in the early 20th century was life saving for people suffering from infectious diseases [1]. Despite the landmark progress made in drug development, studies have shown there is progressive resistance to conventional antibiotics by most of the hospital-acquired pathogenic bacteria classified as ESKAPE [2] organisms, including *Enterococcus faecium* [3,4], *Staphylococcus aureus* [5,6,7,8], *Klebsiella pneumoniae* [9,10,11,12], *Acinetobacter baumannii* [13,14,15,16], *Pseudomonas aeruginosa* [17,18,19,20,21] and *Enterobacter* sp. [22,23]. A World Health Organization (WHO, Geneva, Switzerland) report in 2019 concluded that, if the current trend is not averted, drug-resistant diseases could lead to the death of 10 million people each year by 2050 [24,25].

*P. aeruginosa* relies on quorum sensing-based gene regulations as a major contributor to their pathogenesis and drug resistance [26]. Quorum sensing (QS) is a cell-to-cell communication system used by many microorganisms to coordinate gene expression at the population level [27]. This communication system involves the secretion of chemical signaling molecules and once a sufficient concentration of signal molecules is achieved, this induces the expression of genes involved in a number of phenotypes, including biofilm formation [26,28], virulence factor production [29,30] and drug resistance mechanisms [31]. Specifically, *P. aeruginosa* possesses four QS systems, such as LasI/LasR, Rhll/RhlR, pseudomonas quinolone signal (PQS) and the integrated quorum-sensing signal (IQS) [32,33,34]. LasI/LasR and Rhll/RhlR are acyl homoserine lactone (AHL)-dependent QS systems in *P. aeruginosa* [35]. The interactions of the AHLs, namely *N*-3-oxododecanoyl homoserine lactone and *N*-butyryl-homoserine lactone, produced by these systems with the respective regulatory proteins, LasR and RhIR, activate the transcription of nearly 10% of about 300 genes in *P. aeruginosa* [36].

The QS-induced expression of *lasB* and *rhlA* genes in *P. aeruginosa* encodes the production of elastase and rhamnolipids, respectively [37,38,39]. Elastase LasB, an extracellular zinc metalloprotease, facilitates extensive host colonization [40], suppresses the innate immune system [41] and causes damage to the host tissues [42,43] The elastase gene in *P. aeruginosa* was first discovered by Mandl and colleagues in 1962 [44] and later confirmed to be the major encoding gene for elastolytic activity responsible for the pathogenesis in *P. aeruginosa*-infected tissues [45]. This elastase enzyme has been identified as a possible therapeutic target to attenuate the mechanism of continuous virulence and progression of the disease by *P. aeruginosa.* Rhamnolipids, which are amphipathic glycolipids and encoded by the *rhlAB* operon and *rhlC*, play multiple functions in the maturation and preservation of biofilms by assisting in the formation of microcolonies and extracellular polymeric substances that are embedded in the bacterial community [46,47]. A study conducted by Davies and co-workers revealed a correlation between the QS signaling system and biofilm development in *P. aeruginosa* pathogenesis [32]. The biofilm mode of development serves as a survival strategy for pathogenic microorganisms to increase antibacterial resistance and cause severe systemic infections [48,49].

The interference of the QS system in pathogenic bacteria with the use of small molecules as potential inhibitors represents an attractive target for the disruption of biofilm formation, attenuating virulence factors as well as combating microbial resistance [25]. Moreover, studies revealed that the combination of a quorum sensing inhibitor (QSI) with an antibiotic resulted in the attenuation of biofilm formation and improvement in antibiotic penetration into a pathogenic bacterial cell, while reducing virulence factor production [50]. For instance, the use of antibiotics, such as ciprofloxacin, tobramycin and colistin, with a synthetic QSI, *N*-(2-pyrimidyl)butanamide, reduced biofilm formation and improved antibiotic efficacy in cystic fibrosis (CF) lung infection [51]. As *N*-(2-pyrimidyl)butanamide interfered with QS, it caused the bacteria to transition from biofilm to a planktonic state, allowing antibiotics to kill the microbes. Currently, several potent QSIs, including ET37, linolenic acid and a 6-gingerol analog, are being explored in virulence attenuating combination therapy with known antibiotics for the treatment of CF lung infection [50,52].

Psammaplin A (**1**) and its derivatives have attracted much attention due to their significant therapeutic activities [53,54,55,56,57]. In particular, psammaplin A possesses anticancer properties against various cancer cell lines, such as triple-negative breast, doxorubicin-resistant human breast, colon, ovarian, lung, bone, brain, skin, and central nervous system cancer cell lines [53]. In addition, this molecule displayed antibacterial activity against pathogenic bacterial strains, including *Staphylococcus aureus*, methicillin-resistant *Staphylococcus aureus* as well as suppressing *Vibrio vulnificus*-induced cytotoxicity in the in vitro and in vivo studies [55,56]. Moreover, its antibacterial activities are attributed to the molecule’s inhibition of bacterial DNA gyrase and DNA synthesis [55].

The current study addresses the urgent need for new structural templates as quorum sensing inhibitors for the treatment of pathogenic bacterial infections through the use of various QS-based bioassay platforms. In our search from marine sources for novel quorum sensing antagonists, we test psammaplin A (**1**) and bisaprasin (**2**) for quorum sensing inhibitory activity. These two compounds, previously isolated alongside other psammaplins [55,58,59,60] and bromotyrosine compounds from the methanolic extract of the marine sponge, *Aplysinella rhax*, collected from the Fiji Islands, are subjected to QS inhibitory screening, anti-elastase enzymatic and anti-biofilm formation assays. The other metabolites are not isolated in a sufficient quantity for the current study. Psammaplin A (**1**) is composed of two modified amino acids: a bromotyrosine, containing an oxime group, and cysteamines that form the disulfide bridge [58].

In this paper, we report the inhibitory activities of psammaplin A (**1**) and its biphenylic dimer, bisaprasin (**2**), on QS-regulated genes expression, QS-induced LasB elastase production and biofilm formation in *P. aeruginosa* PAO1.

## 2. Results and Discussion

### 2.1. Isolation and Structure Elucidation

**Figure 1:**
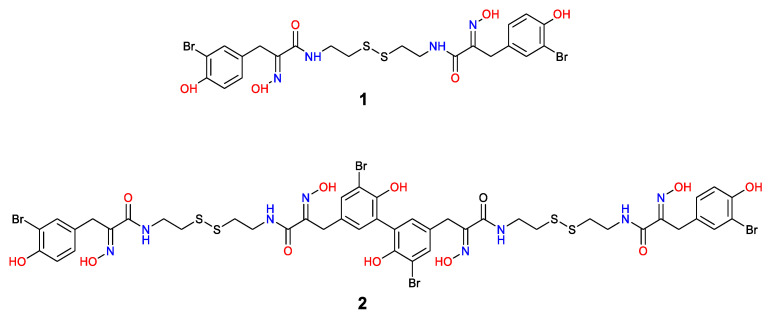
Structures of psammaplin A (**1**) and bisaprasin (**2**).

The marine sponge extract was partitioned between water and dichloromethane (50% *v*/*v*) using a modified Kupchan method as previously described [57,61] and the CH_2_Cl_2_ fraction was further fractionated using reversed-phase solid-phase extraction (SPE). The resulting 100% SPE fraction was purified on reverse-phase HPLC to yield **1** (5.4 mg) and **2** (5.6 mg). The structures of psammaplin A (**1**) and bisaprasin (**2**) (Figure 1) were determined based on the interpretation of their experimental 1D and 2D NMR and HRESIMS data (Figures S1–S10), which were comparable with those previously described [54,58,59,60,62].

### 2.2. Inhibition of the Quorum Sensing Systems of P. aeruginosa

**Figure 2:**
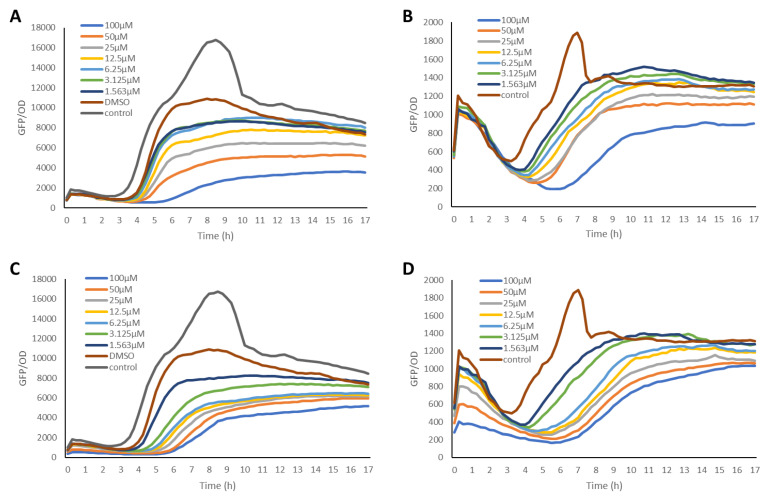
Dose–response curves of psammaplin A (**1**) incubated with P. aeruginosa PAO1 *lasB-gfp*(ASV) (**A**) and *rhlA-gfp*(ASV) (**B**) strains, while (**C**,**D**) are the dose–response curves of bisaprasin (**2**) incubated with *P. aeruginosa* PAO1 *lasB-gfp*(ASV) and *rhlA-gfp*(ASV) strains, respectively.

**Figure 3:**
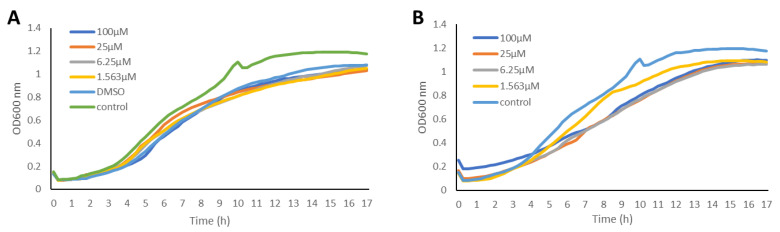
The growth curve (OD600) of the biosensor strain PAO1 *lasB-gfp*(ASV) incubated with psammaplin A (**1**) (**A**) and bisaprasin (**2**) (**B**) at four different concentrations ranging from 1.563 μM to 100 μM.

The anti-QS activities of the bromotyrosine-containing compounds **1** and **2** (Figure 1) were evaluated for their ability to inhibit QS-controlled green fluorescent protein (GFP) expression using the biosensor strains *P. aeruginosa* PAO1, where the *lasB* and *rhlA* promoters were fused to an unstable *gfp*(ASV) [63,64]. Elastase, encoded by the *lasB* gene, is a virulence factor that is controlled by LasR [65], while the RhlA enzyme is encoded by the *rhlA* gene and is involved in rhamnolipid and polyhydroxyalkanoate production [66]. It has been shown that the RhlR of the *rhl* QS system is required for the expression of the *rhlA* gene. In these reporter strains, the production of the green fluorescent protein (GFP) is indicative of QS induction. QS inhibitor activity is reflected in a reduction in GFP production relative to the control. The GFP expression was measured in relative fluorescence units and normalized by dividing the GFP values by the corresponding OD600 value measured at that time point. Both psammaplin A and bisaprasin were revealed to inhibit LasR-controlled GFP expression in a dose-dependent manner (Figure 2) without affecting bacterial cell growth as monitored by its OD600 absorbance (Figure 3). The bacterial growth curves exhibited a typical log and stationary phase. As expected, the control strain showed the highest GFP-per-OD values, which refer to the PAO1 strains grown without the test compounds. The experiment was performed in biological triplicates. QS inhibition activity was determined at a concentration range of 100 μM to 1.563 μM for compounds **1** and **2** (Figure 2).

**Figure 4:**
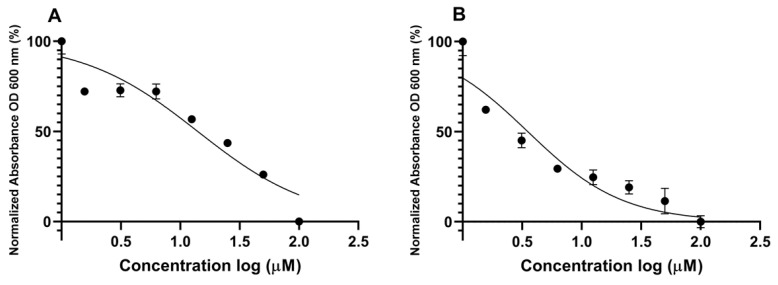
Log IC_50_ curves of psammaplin A (**1**) (**A**) and bisaprasin (**2**) (**B**) incubated with *P. aeruginosa* PAO1 *lasB-gfp*(ASV).

The slope of the curve for each QSI was calculated based on its respective dose–response curves (from Figure 2) and plotted against the log inhibitor concentration. The slope relates to the biosynthesis rate of GFP due to acyl homoserine lactone induction. The half-maximal inhibitory concentration (IC_50_ values) for compounds **1** and **2** were calculated from their dose–response curves by using Graphpad Prism 6 software package (Figure 4). The results were obtained in a low micromolar range for **1** and **2**, with bisaprasin showing the most significant inhibition with IC_50_ values at 2.41 μM and 3.53 μM in the *P. aeruginosa* PAO1 *rhlA-gfp* and *lasB-gfp* biosensor strains, respectively (Table 1).

By comparing the overall inhibition, **2** showed similar inhibition in both *P. aeruginosa* PAO1 *rhlA-gfp* and *lasB-gfp* expression, while **1** exhibited specific inhibition on *P. aeruginosa* PAO1 *rhlA-gfp* expression. The differential inhibition observed in the two molecules could be due to the dimeric nature of bisaprasin (**2**) having a higher number of hydrogen bond donors and acceptor functional groups. Regardless of the differential QSI activities, both psammaplin A and bisaprasin contain bromotyrosine as well as the unique oxime moieties, which could contribute to the observed QSI activity in the bacterial biosensor strains. It has been reported that the presence of the oxime functionality is important for the isoform selectivity of psammaplin A on histone deacetylases [67]. From the compound library, maintained at the laboratory of T.L.T., a bromotyrosine analog, hemifistularin 3, was previously screened for QSI properties, but showed weak inhibition with no clear dose-dependent response based on the PAO1 *lasB-gfp* and *rhlA-gfp* biosensor strains (unpublished data, Table 1 and Figure S11). For instance, the inhibition of **1**, **2** and hemifistularin 3 on PAO1 *lasB-gfp* expression when tested at 100 μg/mL was 85.4, 80.1 and 31.4%, respectively (Table 1). The sponge-derived hemifistularin 3 contains a spirocyclohexadienyl-isoxazoline ring unit and lacks the free oxime moiety. Based on this preliminary comparison with hemifistularin 3, the observed QSI activity of **1** and **2** could be attributed to the oxime unit. However, comparisons of the structures of psammaplin A and bisaprasin with that of hemifistularin show several other differences and further work would be required to confirm the importance of the oxime unit for QSI activity. In addition, synthetic analogs, generated via modifications of these compounds, including reduction of the disulfide bonds and aromatic substitution pattern, could be analyzed to determine the pharmacophore.

Several marine-derived bromotyrosine-related compounds have been reported to have QSI activity. A recent study by Tintillier and co-workers revealed the QS inhibitory activity of a series of bromotyrosine compounds, namely aplyzanzine E and two 2-aminoimidazolic derivatives (e.g., purealidin A), isolated from the Polynesian sponge, Pseudoceratina n. sp. [68]. These molecules inhibited QS of marine bacterial Vibrio harveyi BB120 strain at 5 μg/mL and they delayed the onset of luminescence by up to 44.1 min. Psammaplin A and bisaprasin share some chemical features with these QSI active compounds, such as the presence of bromotyrosine and phenolic moiety, which could be important for QSI activity. Moreover, the occurrence of bromine atoms and/or a phenol functional group is present in previously reported, known QS inhibitors from marine sources. To the best of our knowledge, the QSI activity of psammaplin A and bisaprasin on *P. aeruginosa* biosensor strains is described in this paper for the first time.

Based on the same bacterial biosensor strains used in this study, structurally diverse natural products have been uncovered previously to possess anti-QS properties, some of which have similar activities compared with psammaplin A and bisaprasin. An early study conducted on a garlic-derived compound, ajoene, revealed its IC_50_ values at 15 μM and 50 μM in the PAO1 *lasB-gfp* and *rhlA-gfp* reporter strains, respectively [69]. Subsequently, 25 disulfide bond-containing analogues were synthesized and a benzothiazole derivative was identified to reduce QS-regulated virulence factors and successfully inhibit *P. aeruginosa* infection in a murine model of implant-associated infection [70]. A total of 5 structurally unrelated QSIs were also identified from a natural-derivative database comprising 3040 natural molecules. The IC_50_s of these five compounds range from 0.64 to 3.65 μM based on the PAO1 *lasB-gfp* reporter strain [71]. Trikoramides, novel cyanobactins isolated from the marine cyanobacterium, *Symploca hydnoides*, were recently reported to possess QSI activity based on PAO1 *lasB-gfp* and *rhlA-gfp* strains. Of this series, the Br-containing trikoramide D exhibited moderate to significant dose-dependent quorum sensing inhibitory activities against PAO1 *lasB-gpf* and *rhlA-gfp* bioreporter strains with IC_50_ values of 19.6 µM and 7.3 µM, respectively [72]. In a nutshell, the reporter strains used in this study are an effective screening platform for the uncovering of structurally diverse QSIs with potential therapeutic usage in treating *P. aeruginosa* infections.

Since a wide range of *P. aeruginosa* virulence factors is controlled by quorum sensing regulatory proteins, such as LasR, RhlR and PqsR, their inhibition has been targeted as a viable solution to the control of quorum sensing-mediated infections [73]. A number of natural products and synthetic molecules having significant inhibitions against these regulatory proteins have been identified [73]. For instance, the plant-derived natural product, coumarin, was recently revealed as a potent inhibitor of several quorum sensing-related proteins, including AHL syntheses, LasR, RhlR and PqsR, via molecular docking simulations [74]. It has been proposed that its anti-QS property is due to the inhibition of signal molecule synthesis, the antagonization of QS-regulatory proteins and the blocking of receptor proteins. A series of 55 synthetic analogs based on the chemical structure of another natural product, 6-gingerol, was recently evaluated for its QSI activity [75]. From the study, an alkynyl ketone analog was revealed to have selective RhlR antagonism over LasR and PqsR, strong inhibition of biofilm formation as well as reduced production of virulence factors in *P. aeruginosa.* Furthermore, a number of synthetic QSIs, which function as useful probes for mechanistic studies against QS regulatory proteins, could potentially be explored further as potential drugs due to their predicted desirable physicochemical properties, including lead likeness, Lipinski rule and LogP values [73]. As a future work, it would be interesting to assess psammaplin A and bisaprasin in other P. aeruginosa biosensor strains and if they are specific inhibitors of these quorum sensing regulatory proteins.

### 2.3. Effects of Psammaplin A and Bisaprasin on Elastase Production in P. aeruginosa

Elastase, a major virulence factor of *P. aeruginosa*, is encoded by the lasB gene and, as such, its production is regulated by the las QS system [76]. Consequently, we hypothesize that the compounds, which showed significant QSI activity when tested on the *P. aeruginosa* PAO1 *lasB-gfp* biosensor strain, would also show inhibition of elastase production. A standard enzymatic elastase assay was used to evaluate whether compounds **1** and **2** could inhibit the elastase production of *P. aeruginosa* cultures at two concentrations of 50 μM and 100 μM (Figure 2). 

**Figure 5:**
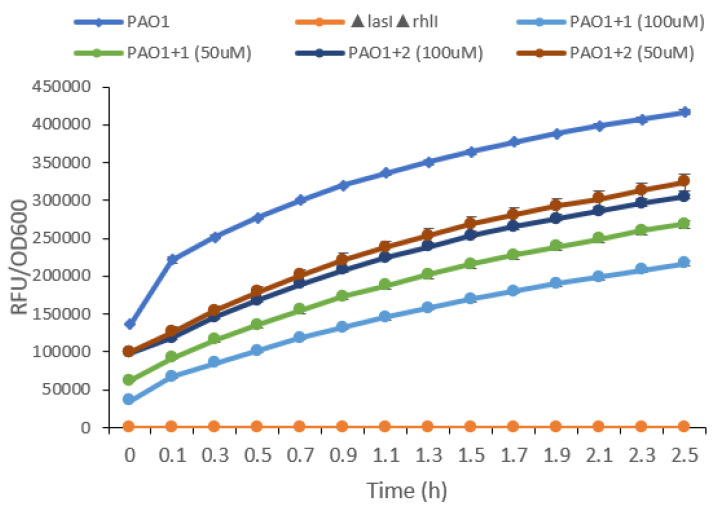
Effects of psammaplin A (**1**) and bisaprasin (**2**) on the elastase activities of *P. aeruginosa* cultures. The elastase activity of *P. aeruginosa* culture supernatants was measured using the EnzChekElastase assay kit (Invitrogen). Fluorescence was recorded every 6 min for 2.5 h by using a Tecan Infinite 200 Pro plate reader (excitation at 490 nm, emission at 520 nm). The *P. aeruginosa* PAO1 Δ*lasI*Δ*rhlI* strain and DMSO served as controls.

Both compounds exhibited a dose-dependent inhibition of elastase activity within a 2.5 h cultivation period (Figure 5). One noteworthy point is psammaplin A at 50 μM had a higher inhibition than bisaprasin at 100 μM. Psammaplin A, evidently when administered at 100 μM, the concentration at which the compound exhibited the highest inhibition, can reduce the amount of elastase expression by approximately 50% relative to the wild-type strain. However, neither compound was able to abolish the production of elastase completely at 100 μM by matching the levels of a *P. aeruginosa* PAO1 Δ*lasI*Δ*rhlI* mutant strain. This *P. aeruginosa* PAO1 Δ*lasI*Δ*rhlI* mutant is deficient in quorum sensing and is used as a negative control in the assay. The results from the elastase assay were not consistent with the PAO1 *lasB-gfp* biosensor assay since bisaprasin was found to be more active in the former assay. This could be due to the involvement of a complex QS regulatory system in the expression of the elastase enzyme. Studies revealed that *lasB* expression can be induced through the activation of other systems, such as RhlR and PqsR, regardless of the functional status of LasR, which is the primary regulator of *lasB* expression [77].

A number of compounds have been shown to prevent/reduce the production of elastase. For instance, Tan and co-workers reported a molecule, 5-imino-4,6-dihydro-3H-1,2,3-triazolo[5,4-d]pyrimidin-7-one, when added to *P. aeruginosa* PAO1 at 50 and 100 μM could almost abolish elastase production within a 2 h period [71]. In another study, two synthetic itaconimide-related analogs, when tested at a lower concentration of 10 μM, were able to reduce elastase production by almost half as compared to the wild PAO1 strain [78]. Similarly, the inhibitory effect of falcarindiol, a polyyne isolated from a Chinese herb, *Notopterygium incisum*, on virulence factors was dose dependent, ranging from 2.5 μM to 20 μM. The production of elastase in PAO1 by this molecule was inhibited by 37.17% when tested at 20 μM [79].

Taken together, the QSI and elastase results indicate that psammaplin A (**1**) and bisaprasin (**2**) have potential as QS inhibitors. The list of QS inhibitors from the marine source is populated by our findings since the first discovery of the two brominated furanones QS inhibitors that were isolated from the *D. pulchra* [80]. Based on these data, we surmise that psammaplin A and bisaprasin downregulate the expression of elastase through the inhibition of the *lasB* QS system of *P. aeruginosa*. The greater reduction in elastase activity seen in samples treated with psammaplin A compared to bisaprasin suggests that psammaplin A might be suitable to progress to in vivo infection control studies.

### 2.4. Evaluation of Psammaplin A and Bisaprasin against Biofilm Formation in P. aeruginosa

**Figure 6:**
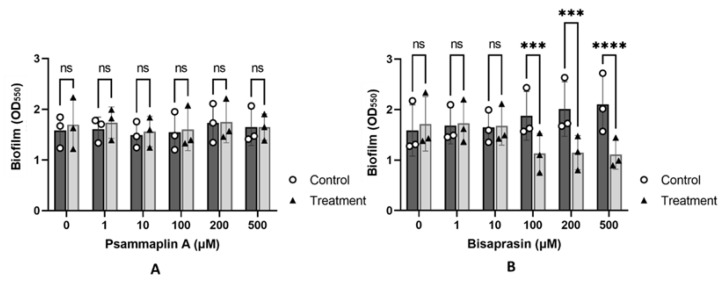
(**A**,**B**) show the effects of 0–500 µM of psammaplin A (**1**) and bisaprasin (**2**) on *P. aeruginosa* PAO1 biofilm formation, respectively. Each data point represents the average of two technical replicates. Error bars indicate the standard deviation of the mean. *p*-values were derived from multiple comparisons between control and treatment groups following two-way ANOVA, with ***—<0.001, ****—<0.0001. ns= not significant.

In addition to the regulation of virulence genes, QS play a role in the regulation of biofilm formation and development in *P. aeruginosa* [81]. Mutants of QS-related genes are known to form reduced or morphologically distinct biofilms [26,82]. In addition, several studies have shown that QS inhibitors are associated with the inhibition of biofilm formation [83,84]. As such, we evaluated the ability of compounds **1** and **2** to inhibit biofilm formation in *P. aeruginosa* PAO1 (Figure 6).

Psammaplin A (**1**) had no effects on PAO1 biofilm formation across all concentrations (Figure 6A). In contrast, bisaprasin (**2**) was able to reduce the extent of biofilm formation at higher concentrations at or above 100 µM (Figure 6B). This reduction in biofilm formation was not due to the toxicity of **1** or **2**, as the compounds were used below their minimum inhibitory concentration (MIC > 1024 µM, highest concentration evaluated, Figure S12). In comparison, for the same strain, MIC of antibiotics, such as colistin and gentamicin, is <1 ug/mL. The reduction was concentration dependent, with 100, 200 and 500 µM of compound **2** displaying 40, 43 and 46% reduction in PAO1 biofilms, respectively (Figure 6B). This is consistent with QSI assays, which indicated that **2** was relatively more potent than **1**.

However, while biofilm inhibition increased with concentration, it was not proportional to the amount of compound used. This is not unexpected given that other studies on QS inhibitors had indicated that the inhibition of QS activity does not necessarily translate to biofilm inhibition, and some compounds with a high inhibition of QS were found to have a limited effect on biofilm formation [85]. Likewise, while mutations in the *las* system are highly correlated with reduced biofilm formation, some isolates with defective lasR may continue to form weak biofilms. Similarly, *P. aeruginosa* isolates harboring mutations in *rhlR* could also form weak-to-moderate biofilms, suggesting that other pathways may influence biofilm formation [86]. In correlating this with the results from other assays used in the current study, it seems to suggest that, for a compound to exhibit antibiofilm activity, it needs to be relatively active in both the PAO1 *lasB-gfp* and *rhlA-gfp* biosensor strains, as observed in compound **2**. Although psammaplin A was found to have significant QS inhibition in the PAO1 *rhlA-gfp* biosensor strain, its IC_50_ value in the *lasB-gfp* biosensor strain was almost four times lower than bisaprasin. This could be due to the involvement of several QS systems, such as Las and Rhl QS systems, in the regulation and formation of biofilms in *P. aeruginosa* [87]. However, further studies would need to be carried out to validate this initial observation.

Taken together, the molecular basis of these compounds’ QSI activity could be due to their possible inhibition on Las and Rhl QS systems, specifically on LasR and RhlR regulatory proteins. Preliminary molecular docking (based on SwissDock) simulated using the X-ray structure of the *P. aeruginosa* LasR ligand-binding domain (LBD) (PDB ID: 2UV0) with psammaplin A and bisaprasin revealed non-binding of these molecules within the LBD of LasR. Interestingly, when molecular docking was performed using the monomeric (thiol) form of psammaplin A, the molecule posited strongly within the LBD of the protein in a similar way to the native autoinducer, *N*-3-oxo-dodecanoyl-L-homoserine lactone (Figure S13). Previous studies revealed that psammaplin A is a natural prodrug that inhibits class I histone deacetylase [88]. In fact, the monomeric thiol form of psammaplin A was found to be exquisitely potent against HDAC1 in vitro with IC_50_ of 0.9 nM [89]. Based on preliminary molecular docking simulations, we hypothesize that psammaplin A could be reduced to the active monomeric thiol form in the bacterial cell and binds to the LBD of LasR, preventing the binding of the native autoinducer, *N*-3-oxo-dodecanoyl-L-homoserine lactone. As such, the monomeric thiol form of **1** could represent a new small molecule inhibitor of the LasR transcriptional activator protein. Chemical modifications of the thiol monomer, including substitution pattern on the aromatic ring, chain extension and changes to the thiol group, could be explored for their binding interactions at the LasR LBD. Further experimental mechanistic studies on these compounds would need to be carried out to confirm their molecular targets.

To date, less than 20 sponge-derived compounds, displaying various chemical structures, have been identified to possess significant QSI activities against different bacterial QS systems [90]. Early reports of these compounds include manoalide, secomanoalide and manoalide monoacetate, isolated from the marine sponge *Luffariella variabilis*, which exhibited significant QS inhibition using the *lasB*-*gfp*(ASV) biosensor with IC_50_ values of 0.66 μM, 1.11 μM and 1.12 μM, respectively [91]. An investigation of the QSI crude extract of *Leucetta chagosensis* revealed the alkaloid isonaamidine A to display the strongest QSI activity in the *Vibrio harveyi* based AI-2 biosensor [92]. A new γ-lactone, plakofuranolactone, purified from the marine sponge *Plakortis* cf. *lita* showed quorum quenching activity using reporter gene assays for long- and short-chain signals (*E. coli* pSB1075, *E. coli* pSB401 and *C. violeaceum* CV026) [93]. A steriodal sponge-derived compound, siphonocholin, obtained from the red sea sponge *Siphonochalina siphonella* was revealed to significantly reduce the production of the QS regulated virulence functions of CV12472 (violacein) and PAO1. In addition, this steroid significantly decreased the biofilm formation ability of several bacterial pathogens, including PAO1, MRSA, *C. violeaceum* and *Acinetobacter baumannii* [94]. Lastly, several bromine-containing alkaloids, such as hymenialdisin, 3-bromo-4-methoxyphenethylamine, 5,6-dibromo-*N*,*N*-dimethyltryptamine, aplyzanzine E, purealidin A, oroidin, benzosceptrin C, and 4,5-dibromopyrrole-2-carboxamide, have been shown to exhibit strong QSI and/or antibiofilm activities based on various bacterial biosensor strains [68,95,96,97]. The discovery of psammaplin A and bisaprasin in this study adds to the growing number of sponge-derived compounds having significant QSI as well as antibiofilm (particularly for bisaprasin) properties. Moreover, these compounds serve as potential lead compounds for the development of antivirulence agents in the treatment of pathogenic bacterial infections.

## 3. Materials and Methods

### 3.1. General Experimental Procedures

IR spectra were recorded on a PerkinElmer UATR Two (Waltham, MA, USA), model L1600300. Both 1D and 2D NMR data were recorded on a Bruker AVANCE III HD Prodigy TCI cryoprobe (Billerica, MA, USA) at 600 and 150 MHz for ^1^H and ^13^C, respectively. HRESIMS data were obtained using a ThermoScientific LTQ XL/LTQ Orbitrap Discovery (Waltham, MA, USA) coupled to a Thermo instrument Accela HPLC system, and an Agilent 6540 HRESI-TOF-MS (Santa Clara, CA, USA) coupled to an Agilent 1200 HPLC system. Fractionations were carried out on solid-phase extraction columns using C18-E (Phenomenex (Torrance, CA, USA), 55 μm, 70 Å, 2 g/12 mL, giga tubes). Purification was performed using an Agilent 1200 semipreparative HPLC system equipped with binary pump, photodiode array detector (DAD)22, Waters Sunfire reversed-phase column C18 (5 μm, 10 × 250 mm) and Agilent Zorbax C18 (5 μm, 9.4 × 250 mm), and a mobile phase solvent gradient between 95:5% and 20:80% (H_2_O/MeOH).

### 3.2. Sponge Collection and Identification

The sponge sample was collected from the Fiji Islands in December 1997, freeze dried and stored in 4 °C. It was identified as *Aplysinella rhax* by Dr. John Hooper of the Queensland Centre for Biodiversity, Queensland Museum, Australia, as described in a previous publication [54]. A voucher specimen (Voucher number: 9712SD130) is held at the Pacific Regional Herbarium at the University of the South Pacific, Suva, Fiji Islands.

### 3.3. Extraction and Isolation of Psammaplin A (1) and Bisaprasin (2)

The freeze-dried sponge sample (420 g) was macerated for 72 h and subsequently extracted with MeOH (3 × 300 mL), followed by DCM (3 × 200 mL). The combined organic extracts were dried under reduced pressure to yield 1.89 g. The sponge extract (1.89 g) was partitioned following the modified Kupchan liquid–liquid partitioning technique described previously [61]. The liquid partitioning process led to four fractions (*sec*-butanol fraction (0.705 g), methanol fraction (0.390 g), CH_2_Cl_2_ fraction (0.152 g) and hexane fraction (0.19 g), which were dried and weighed. The CH_2_Cl_2_ fraction (0.152 g) was further fractionated on a C-18 SPE using aqueous methanol (25%, 50%, 100% and 100% MeOH with TFA) as the mobile phase yielding two interesting fractions: FD-100% MeOH (80 mg) and FD-50% MeOH (52.6 mg) based on ^1^H-NMR profiles. The fraction FD-100% MeOH was purified on a Sunfire reversed-phase column using a gradient solvent system from 80:20 to 0:100% H_2_O/MeOH as mobile phase in 30 min and the complete process of purification that led to the isolation of compound **1** (5.4 mg) and compound **2** (5.8 mg) alongside other metabolites was detailed in the previous publication [57].

### 3.4. Bacterial Strains

To determine the QSI and anti-biofilm activity of the compounds, the various *P. aeruginosa* monitor strains, listed in Table S3, were used. These monitor strains have their respective promoters fused to an unstable GFP (green fluorescent protein) that has a C-terminal oligopeptide extension containing the amino acids ASV (*gfp*(ASV)). This causes the GFP to be more susceptible to degradation by housekeeping proteases and therefore to have a short half-life. As such, unstable *gfp*(ASV) allows for monitoring of temporal QS-regulated gene expression.

### 3.5. P. aeruginosa Quorum Sensing Inhibition Assays

Compounds **1** and **2** were dissolved in 100% DMSO and mixed with ABTGC medium (AB minimal medium containing 2.5 mg/L thiamine, supplemented with 0.2% (wt/vol) glucose and 0.2% (wt/vol) casamino acids) [98], after which they were added to the first column of wells of a 96-well microtiter plate to obtain a final concentration of 100 μM in a final volume of 200 μL. One hundred microliters of ABTGC medium was then added to the remaining wells in the plate. This was followed by serial 2-fold dilutions of the compounds prepared by adding 100 μL of the preceding compound-containing well to the subsequent one. The final column had no test compound as a control. An overnight culture of the *P. aeruginosa lasB*-*gfp*(ASV) and *rhlA-gfp*(ASV) strains, grown in LB medium at 37 °C with shaking, was then diluted to an optical density at 600 nm (OD600) of 0.2, and 100 μL of bacterial suspension was added to each well of the microtiter plate. Thus, each compound was tested at concentrations ranging from 100 μM to 1.563 μM in a volume of 200 μL/well. The microtiter plate was incubated at 37 °C in a Tecan Infinite 200 Pro plate reader (Tecan Group Ltd., Männedorf, Switzerland). GFP fluorescence (excitation at 485 nm, emission at 535 nm) and cell density (OD600) measurements were collected at 15 min intervals for 17 h.

### 3.6. Elastase Assay

*P. aeruginosa* wild type (PAO1 WT) [86] and elastase-negative mutant (PAO1 Δ*lasI*Δ*rhlI*) [63] were each streak-plated on a LB agar plate and incubated at 37 °C overnight. An individual colony from each plate was cultivated in LB medium at 37 °C, with shaking, overnight. Overnight cultures were diluted in 2.5 mL of ABTGC medium in six different tubes (five tubes for PAO1 WT and one tube for PAO1 Δ*lasI*Δ*rhlI*) to a final optical density at 600 nm of 0.01. Each compound, at their respective concentrations, was supplemented into each of the four tubes containing PAO1 WT. All six tubes were then incubated for 24 h at 37 °C with shaking at 200 rpm. After 24 h, all cultures were centrifuged at 197.568 g for 25 min and 0.4 mL of culture supernatants were sampled from each tube. The elastase activity of the *Pseudomonas aeruginosa* culture supernatants was measured using the EnzChekElastase assay kit (Invitrogen, Waltham, MA, USA). The kit consists of BODIPY fluorophore (FL)-labeled DQ elastin conjugate as a substrate of elastase. The BODIPY FL-labeled DQ elastin conjugate, when cleaved by elastase enzyme, yields highly fluorescent fragments. Fluorescence was recorded every 6 min for 2.5 h using Tecan Infinite 200 Pro plate reader with excitation at 490 nm and emission at 520 nm.

### 3.7. Biofilm Assay Screening

*P. aeruginosa* PAO1 WT was grown in LB medium (244,620, Difco) at 37 °C with 200 rpm shaking. Overnight cultures of PAO1 were subsequently diluted 1:200 in 1 × M9 salts (M6030, Sigma Aldrich, St. Louis, MO, USA) supplemented with 0.4% (wt/vol) glucose. The diluted culture (150 µL) was added to each well of a 96 well plate (167,008, Thermo Scientific, Waltham, MA, USA) and compounds **1** and **2** were added to a final concentration of 1–500 µM. The plates were incubated for 6 h with 180 rpm shaking at 37 °C, following which, the medium was removed, and the biofilm was washed once with 1 × PBS and stained with 0.1% (vol/vol) crystal violet for 10 min. The wells were washed twice to remove excess crystal violet and the remaining crystal violet stains were dissolved in 100% ethanol. Biofilms were quantified by measuring absorbance at 550 nm using a microtiter plate reader (Infinite M200, Tecan, Männedorf, Switzerland). Experiments were performed independently three times. Within each independent experiment, control and treatments were performed in duplicate. Averaged values from each independent experiment were plotted and analyzed using Graphpad Prism 9.1.1 using two-way ANOVA with matched values across rows (matched values of control and treatment groups for each independent experiment) and multiple comparisons of cell mean between columns within each row (comparison of effects of treatment within each concentration).

### 3.8. Molecular Docking

The molecular docking method applied comprises the following procedures: ligand preparation, protein selection, docking and analysis of the results. Docking was performed with the SwissDock Docking Web Service (Available online: http://www.swissdock.ch/ (accessed on 28 February 2022)). Three-dimensional structures of the autoinducer (*N*-3-oxo-dodecanoyl-L-homoserine lactone), psammaplin A, bisaprasin and the monomeric (thiol) form of psammaplin A were either obtained from PubChem database or created on Chem3D and converted to .mol2 files using OpenBabel platform (http://openbabel.org/wiki/Main_Page/ (accessed on 28 February 2022)). The LasR protein structure was retrieved from the Protein Data Bank (PDB) with the reference ID (2UV0). The target + ligand set was considered stable when the values of the binding free energy were lower than −7 kcal/mol. This consideration is based on docking experiments with the known X-ray structure (2UV0) complex of the autoinducer and the monomeric (thiol) form of psammaplin A resulting in binding energies values of −10.81 and −8.15 kcal/mol, respectively. Once the results of the docking were obtained, they were analyzed with UCSF Chimera.

## 4. Conclusions

In this study, the quorum sensing inhibitory potential of the psammaplin-related compounds, psammaplin A (**1**) and bisaprasin (**2**), isolated from the marine sponge *Aplysinella rhax* were evaluated in *P. aeruginosa* PAO1 *lasB-gfp*(ASV) and *rhlA-gfp*(ASV) biosensor strains. Bisaprasin showed significant inhibitory activity in both *P. aeruginosa lasB-gfp* and *rhlA-gfp* biosensor strains, while psammaplin A was more active in the PAO1 *rhlA-gfp* biosensor strain. In addition, these compounds inhibited elastase production, while anti-biofilm formation in *P. aeruginosa* cultures was only observed for bisaprasin. Based on the results obtained in this study, we confirmed that both psammaplin A (**1**) and bisaprasin (**2**) have potential as QS inhibitors. A thorough understanding of the mechanism of action is required to elucidate the inhibitory properties of these marine-derived bioactive agents. We are of the opinion that the mode of action of the psammaplin-type compounds is most likely to be interaction with the QS system of the microorganism and the relatively higher potency of **2** against the QS systems could be due to the dimeric nature of the molecule as compared to **1**.

## Figures and Tables

**Table 1:** Quorum sensing inhibitory activity of psammaplin A (**1**), bisaprasin (**2**) and hemifistularin 3 in the *P. aeruginosa* PAO1 *lasB-gfp* and *rhlA-gfp* biosensor strains.

Compound	IC_50_ (μM)		% Inhibition (100 μg/mL)	
	*lasB-gfp*	*rhlA-gfp*	*lasB-gfp*	*rhlA-gfp*
**1**	14.02	4.99	85.4%	63.3%
**2**	3.53	2.41	80.1%	68.9%
Hemifistularin 3	-	-	31.4%	49.1%

## Supplementary Materials

The following are available online. Figures S1–S5: HRESIMS, ^1^H, HSQC, COSY and HMBC NMR spectra of **1**; Figures S6–S10: Orbitrap –(+)-HRMS, ^1^H, HSQC, COSY, HMBC NMR spectra of **2**; Figure S11: Hemifistularin 3 incubated with *P. aeruginosa* PAO1 *lasB-gfp*(ASV) strain at various concentrations; Figure S12: The growth curve (OD600) of *P. aeruginosa* WT incubated with psammaplin A (1) (A) and bisaprasin (2) (B) at concentrations ranging from 1.0 mM to 1024.0 mM; Figure S13: Molecular docking of the LasR-ligand binding domain (PBD ID: 2UV0) with the native autoinducer, *N*-3-oxo-dodecanoyl-L-homoserine lactone and the monomeric thiol form of psammaplin A; Table S1: NMR data for psammaplin A (**1**); Table S2: NMR data for bisaprasin (**2**); Table S3: *Pseudomonas aeruginosa* strains used in the study. References [63, 64, 99, 100] are cited in the supplementary material

## Sample Availability

Not available.
