# Supplementary material for: Inhibition of the Quorum Sensing System, Elastase Production and Biofilm Formation in Pseudomonas aeruginosa by Psammaplin A and Bisaprasin
Source: Molecules. 2022 Mar 6;27(5):1721. doi: 10.3390/molecules27051721 (PMC8911947; doi:10.3390/molecules27051721)
Supplement: Supplementary file 1 [file molecules-27-01721-s001.zip › molecules-1598446-supplementary.pdf]

Supplementary Information

Inhibition of the Quorum Sensing System, Elastase Production and Biofilm Formation in *Pseudomonas aeruginosa* by Psammaplin A and Bisaprasin

Emmanuel T. Oluwabusola [1,*], Nursheena Parveen Katermeran [2], Wee Han Poh [3], Teo Min Ben Goh [2], Lik Tong Tan [2], Oluwatofunmilayo Diyaolu [4] Jioji Tabudravu [5], Rainer Ebel [4], Scott A. Rice [3,6,7] and Marcel Jaspars [4,*]

[1] DDT College of Medicine,Department of Pharmacy, P.O. Box 70587, Gaborone, Botswana.
[2] Natural Sciences and Science Education, National Institute of Education, Nanyang Technological University, 1 Nanyang Walk, Singapore 637616, Singapore; nursheena92@gmail.com (N.P.K.); bengoh93@yahoo.com.sg (T.M.B.G.); liktong.tan@nie.edu.sg (L.T.T.)
[3] Singapore Centre for Environmental Life Sciences Engineering, Singapore 637551, Singapore; whpoh@ntu.edu.sg (W.H.P.); rscott@ntu.edu.sg (S.A.R.)
[4] Marine Biodiscovery Centre, Department of Chemistry, University of Aberdeen, Aberdeen AB24 3UE, Scotland, UK; r01oad17@abdn.ac.uk (O.D.); r.ebel@abdn.ac.uk (R.E.)
[5] School of Forensic and Applied Sciences, Faculty of Science and Technology, University of Central Lancashire, Preston PR1 2HE, England, UK; jtabudravu@uclan.ac.uk
[6] The School of Biological Sciences, Nanyang Technological University, Singapore 639798, Singapore
[7] The iThree Institute, The University of Technology Sydney, Sydney, NSW 2007, Australia

* Correspondence: emmanuel.oluwa@ddtcollegeofmedicine.com (E.T.O.); m.jaspars@abdn.ac.uk (M.J.); Tel.: +26-775-298-641 (E.T.O.); +44-(0)12-2427-2895 (M.J.)

Table of Content **Page**

Table of Content	Page
**Figures S1-S2:** HRESIMS and ^{1}H NMR spectra of **1**	3
**Figures S3-S4:** HSQC and COSY NMR spectra of **1**	4
**Figures S5-S6:** HMBC of **1** and Orbitrap–(+)-HRMS of **2**	5
**Figures S7-S8:** ^{1}H NMR and HSQC NMR spectra of **2**	6
**Figures S9-S10:** COSY NMR and HMBC NMR spectra of **2**	7
**Figure S11:** Hemifistularin 3 incubated with *P. aeruginosa* PAO1 *lasB-gfp*(ASV) strain at various concentrations	8
**Figure S12:** The growth curve (OD_{600}) of *P. aeruginosa* WT incubated with psammaplin A (**1**) (**A**) and bisaprasin (**2**) (**B**) at concentrations ranging from 1.0 μM to 1024.0 μM	8
**Figure S13.** Molecular docking of the LasR-ligand binding domain (PBD ID: 2UV0) with the native autoinducer, *N*-3-oxo-dodecanoyl-L-homoserine lactone (**A**) and the monomeric thiol form of psammaplin A (**B**)	9
**Table S1:** NMR data for psammaplin A (**1**)	9
**Table S2:** NMR data for bisaprasin (**2**)	10
**Table S3.** *Pseudomonas aeruginosa* strains used in the study	11

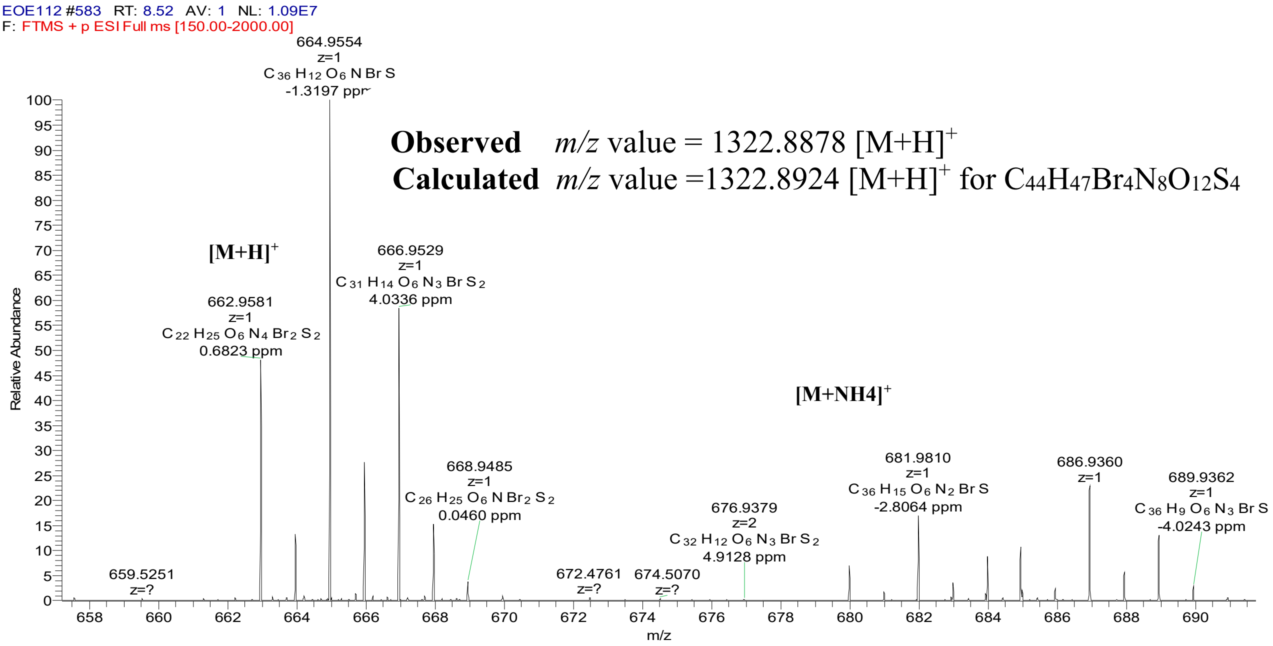

Figure S1. Orbitrap-(+)-HRMS of **1**

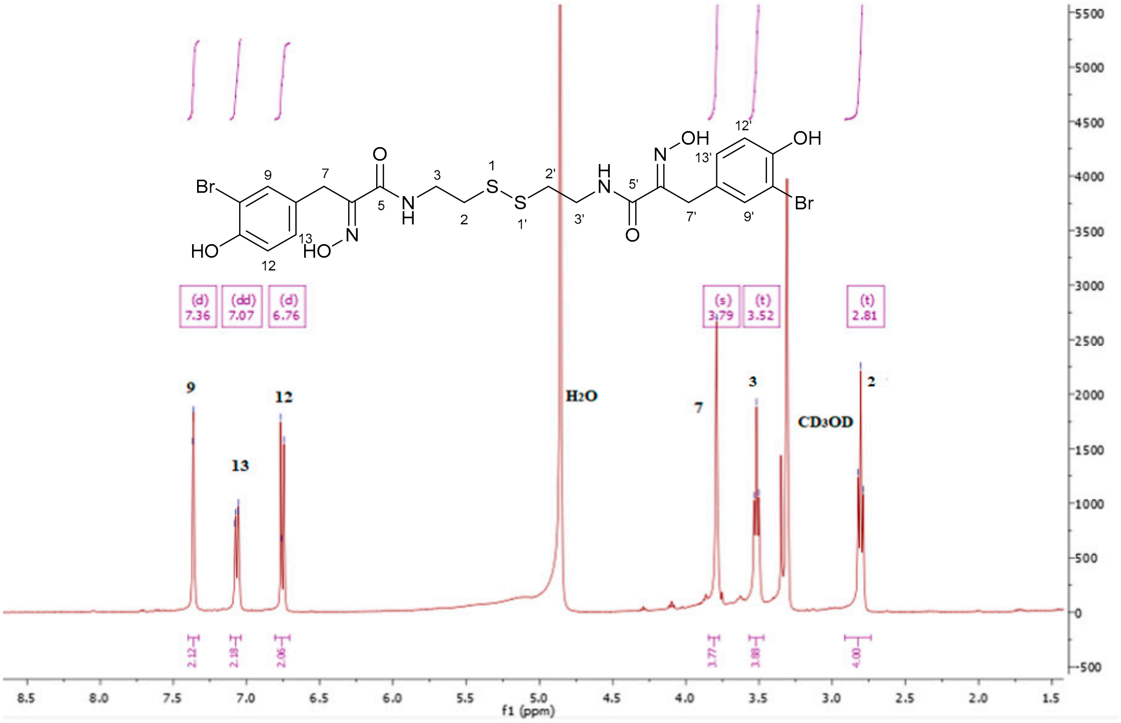

Figure S2. ^{1}H NMR spectrum of **1** at 600MHz in CD_3OD

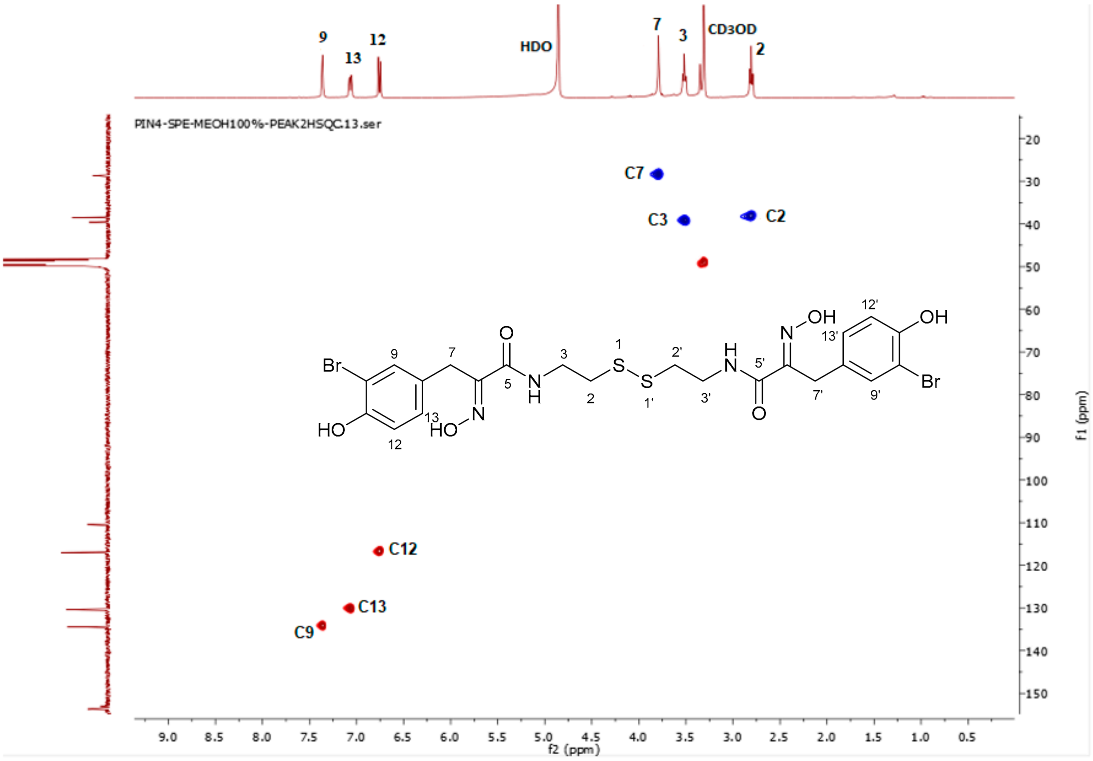

Figure S3. HSQC NMR spectrum of **1** at 600MHz in CD_3OD

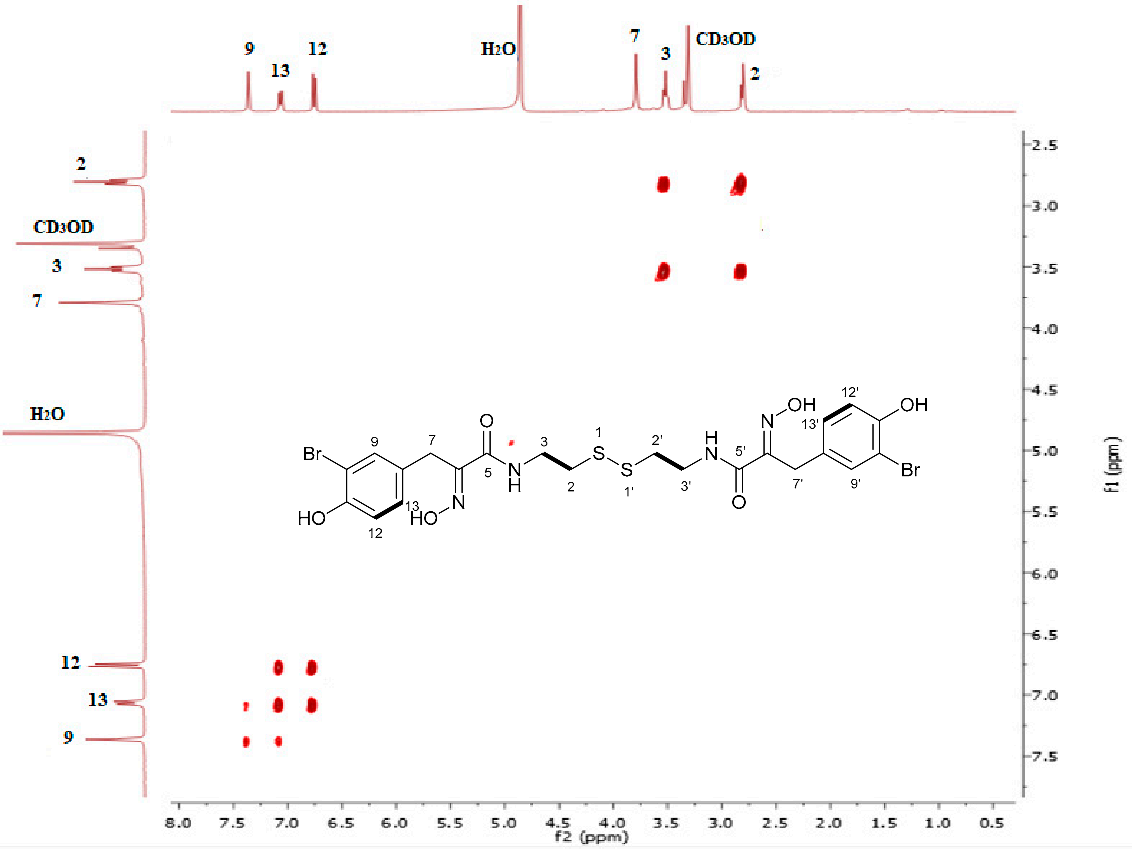

Figure S4. COSY NMR spectrum of **1** at 600MHz in CD_3OD

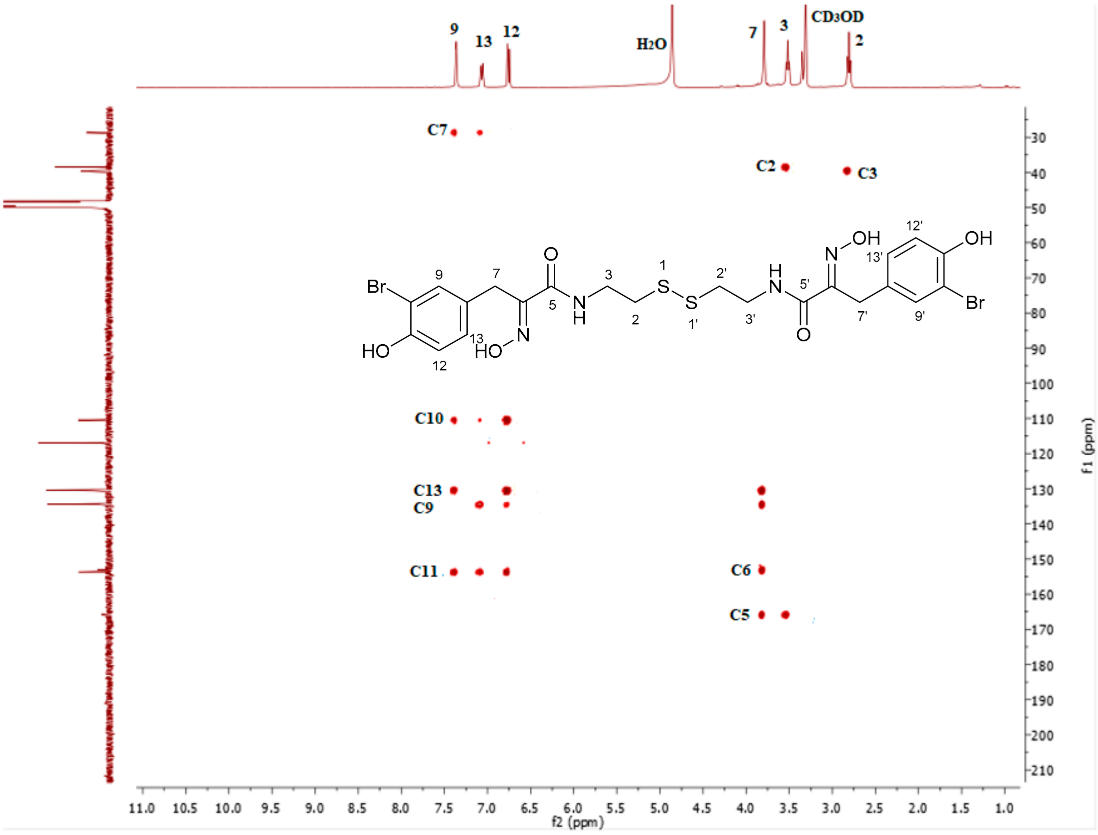

Figure S5. HMBC NMR spectrum of **1** at 600MHz in CD3OD

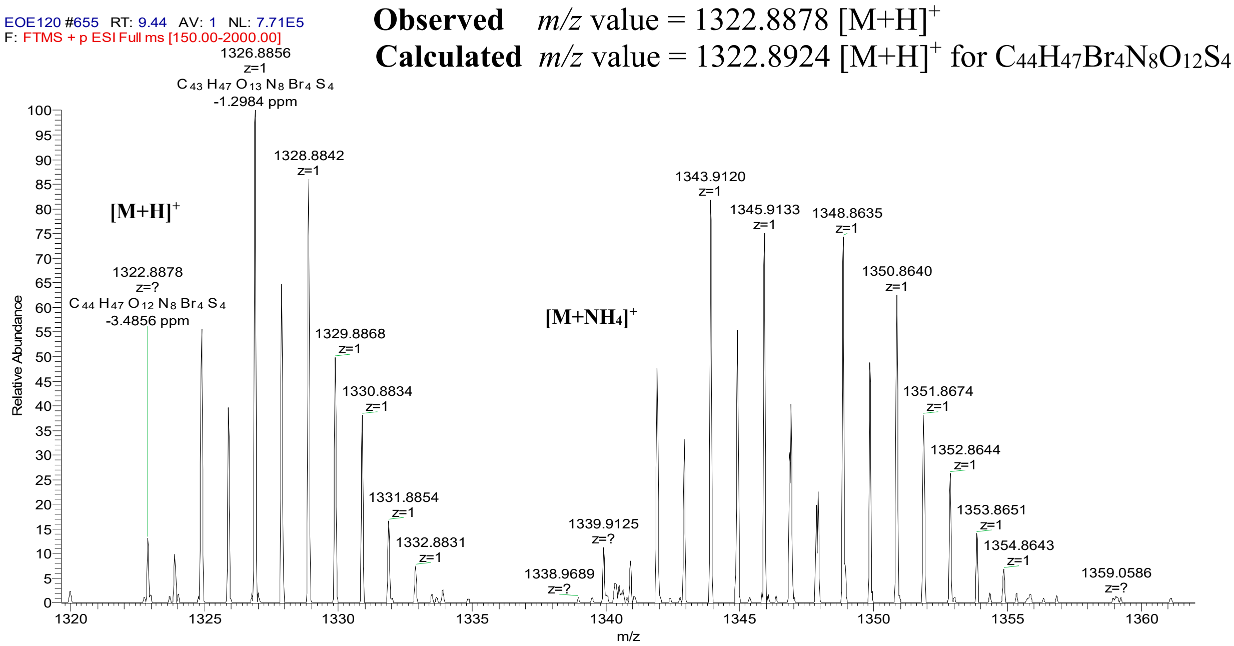

Figure S6. Orbitrap-(+)-HRMS of **2**

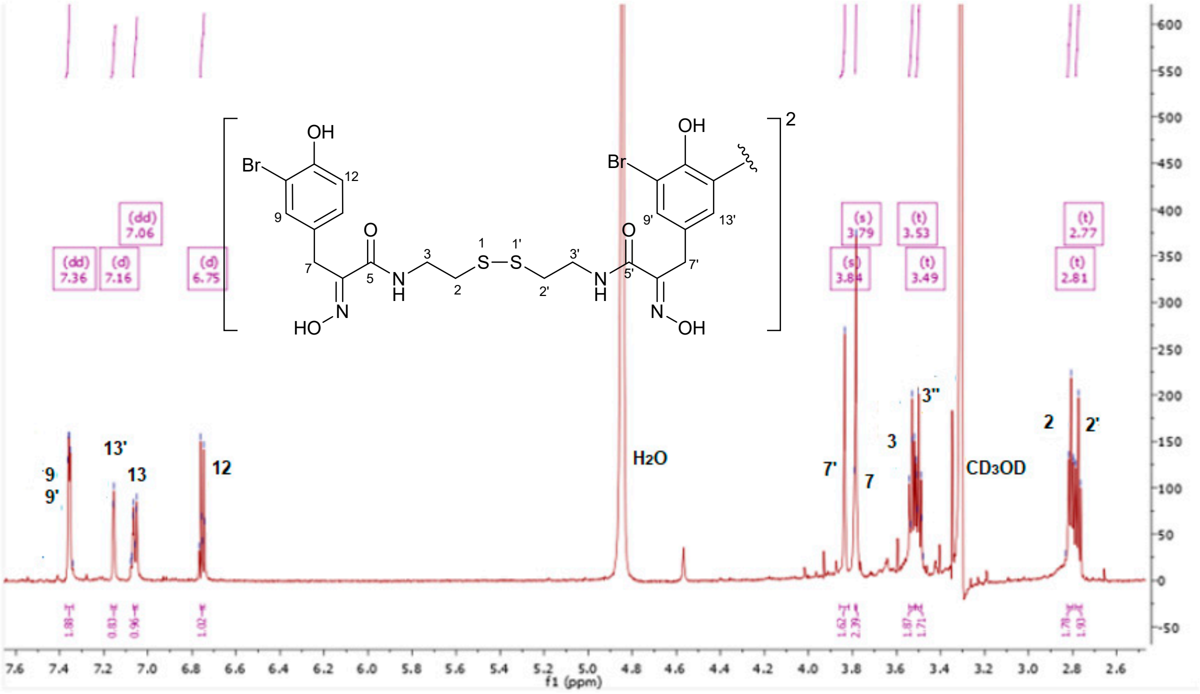

Figure S7. ^{1}H NMR spectrum of **2** at 600MHz in CD_3OD

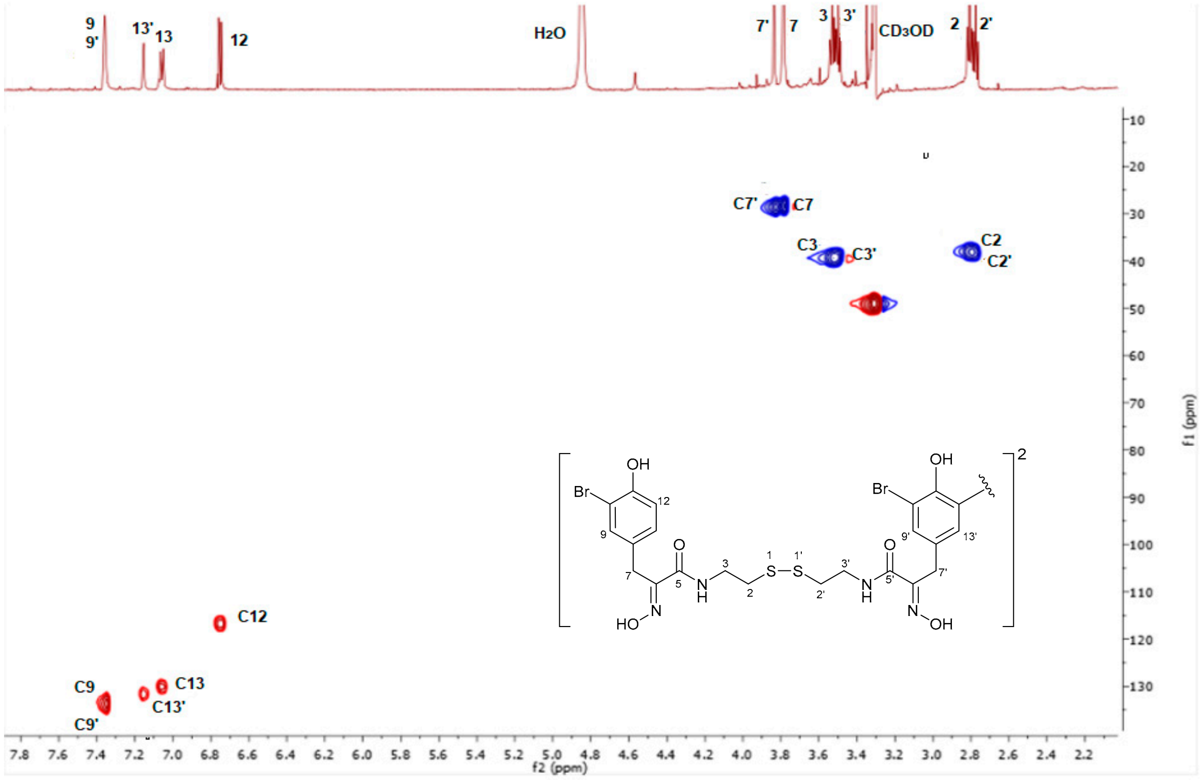

Figure S8. HSQC NMR spectrum of **2** at 600MHz in CD3OD

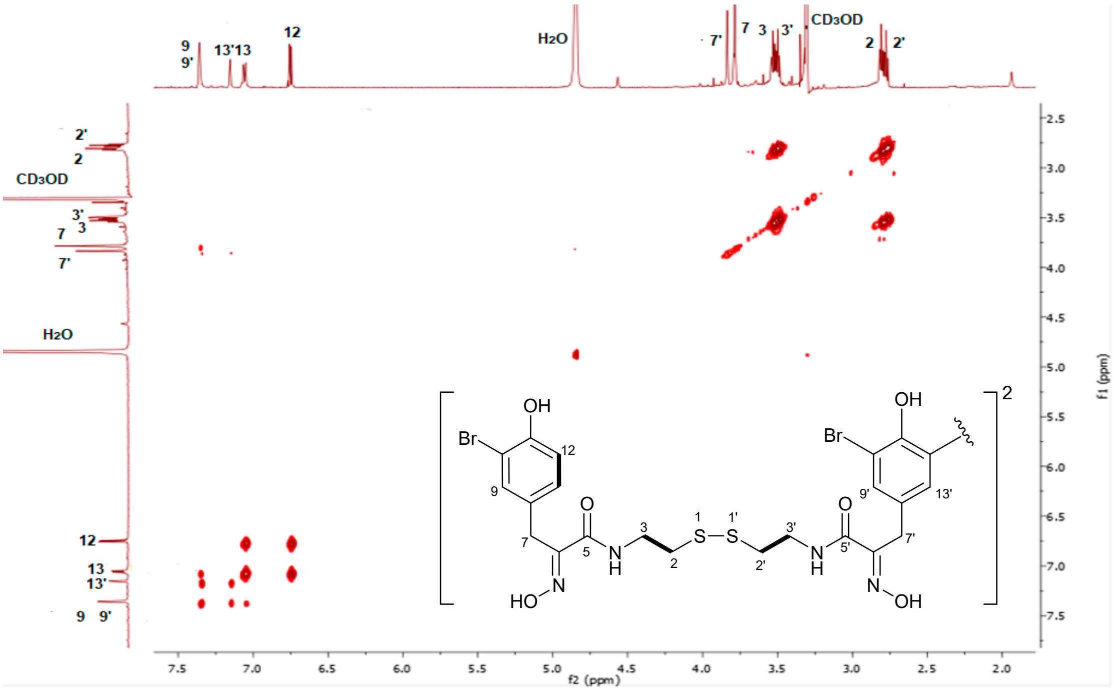

Figure S9. COSY NMR spectrum of **2** at 600MHz in CD_3OD

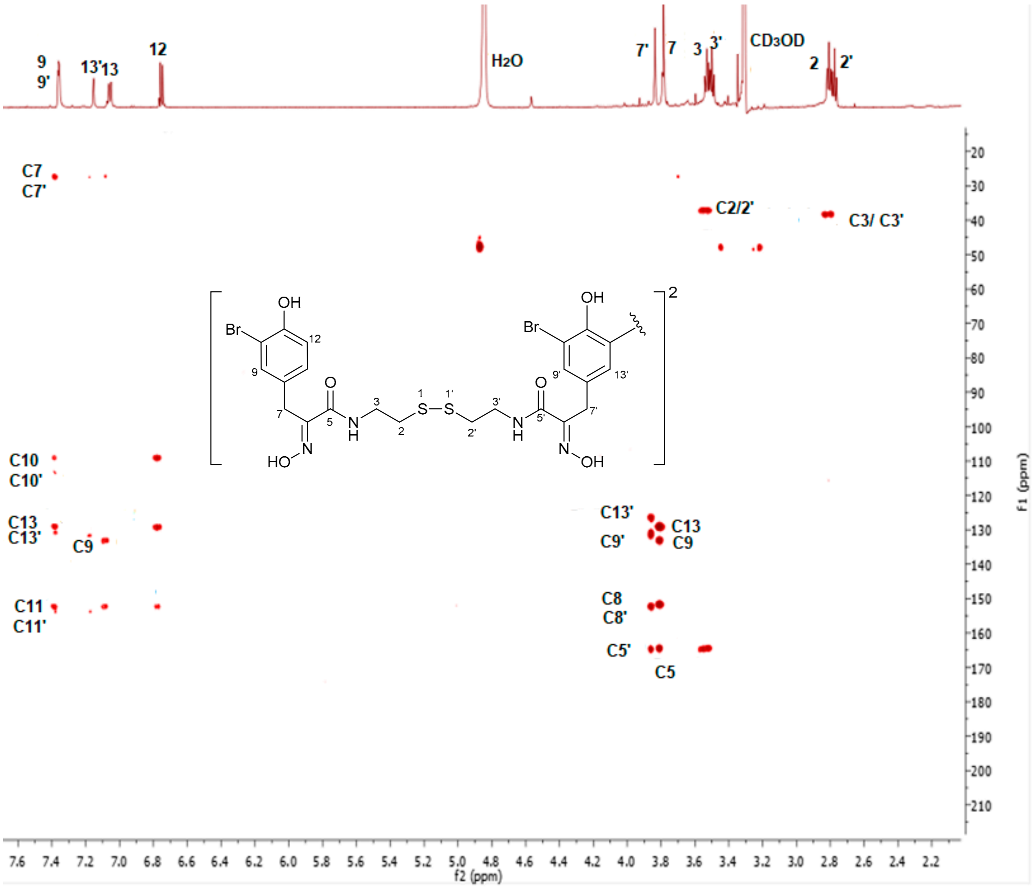

Figure S10. HMBC NMR spectrum of **2** at 600MHz in CD_3OD

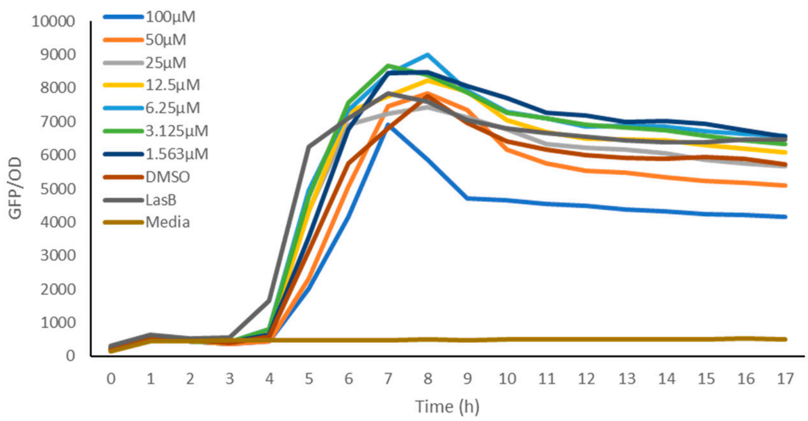

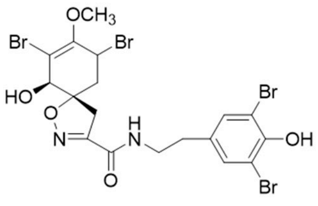

Hemifistularin 3

Figure S11. Hemifistularin 3 incubated with *P. aeruginosa* PAO1 *lasB-gfp*(ASV) strain at various concentrations

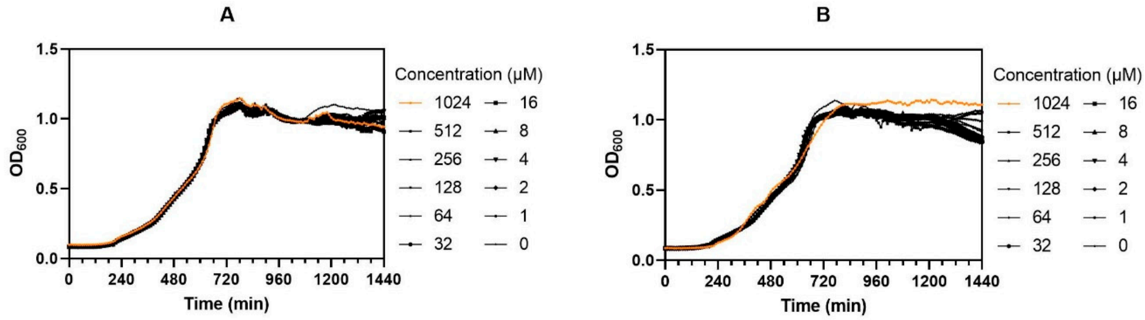

Figure S12. The growth curve (OD600) of *P. aeruginosa* WT incubated with psammaplin A (**1**) (**A**) and bisaprasin (**2**) (**B**) at concentrations ranging from 1.0 µM to 1024.0 µM

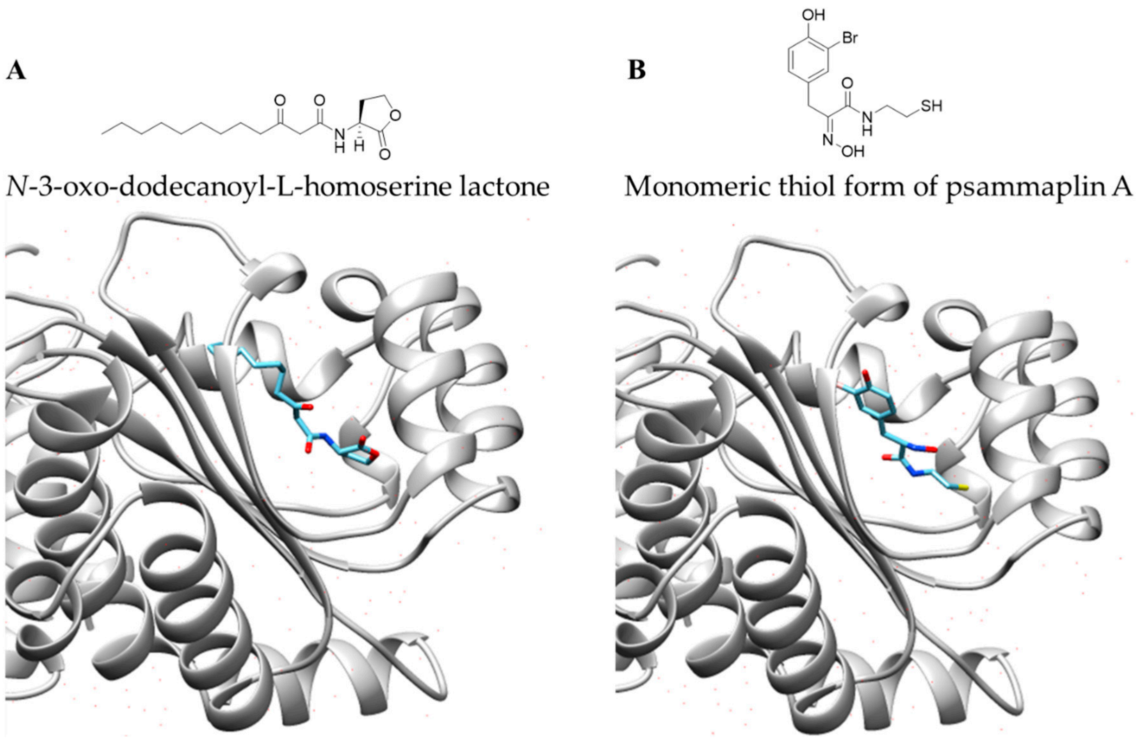

Figure S13. Molecular docking of the LasR-ligand binding domain (PBD ID: 2UV0) with the native autoinducer, *N*-3-oxo-dodecanoyl-L-homoserine lactone (**A**) and the monomeric thiol form of psammaplin A (**B**)

Table S1 NMR data for Psammaplin A (**1**)

**Position**	**δ_C, Type**	**δ_H, mult. (*J* in Hz)**	**HMBC (H→C)**
2, 2’	38.4, CH_2	2.81, t (6.0)	3/3’
3, 3’	39.5, CH_2	3.52, t (6.0)	2/2’, 5/5’
5, 5’	165.8, C		
6, 6’	152.1, C		
7, 7’	28.6, CH_2	3.79, s	5/5’, 6/6’,9/9’ 13/13‘
8, 8’	130.6, C		
9, 9’	134.3, CH	7.36, d 2.0)	7/7’, 10/10’, 11/11’, 13/13’
10, 10’	110.4, C		
11, 11’	153.7, C		
12, 12’	116.8, CH	6.76, d (8.4)	9/9’, 10/10’, 11/11’, 13/13’
13, 13’	130.6, CH	7.07, dd (8.4,2.0)	7/7’, 9/9’, 10/10’, 11/11’
NMR solvents used for **1** was CD_3OD at 600 MHz.			

Table S2 NMR data for Bisaprasin (**2**)

**Position**	**δ_C, Type**	**δ_H, mult.(*J* in Hz)**	**HMBC (H → C)**
2, 2’	38.4, CH_2	3.52, t (6.0)	3/3’
3, 3’	39.5, CH_2	3.58, t (6.0)	2/2’, 5/5’
5, 5’	165.8, C		
6, 6’	153.8, C		
7, 7’	28.6, CH_2	3.79, s	5/5’, 6/6’,9/9’ 13/13‘
8, 8’	130.6, C		
9, 9’	134.3, CH	7.36, d (1.6)	7/7’, 10/10’, 11/11’, 13/13’
10 , 10’	110.4, C		
11,11’	155.7, C		
12, 12’	116.8, CH	6.75, d (8.0)	9/9’, 10/10’, 11/11’, 13/13’
13, 13’	130.6, CH	7.07, dd (8.0,1.6)	7/7’, 9/9’, 10/10’, 11/11’
2”,2”’	38.7, CH_2	2.77, t (6.0)	3’’/3’’’
3”,3”’	39.8, CH_2	3.49, t (6.0)	2’’/2’’’, 5’’/5’’’
5”, 5”’	166.1, C		
6”, 6”’	153.5, C		
7”, 7”’	28.6, CH_2	3.88 s	5’’/5’’’, 6’’/6’’’, 9’’/9’’’, 13’’/13’’’
8”, 8”’	128.3, C		
9”, 9”’	133.4, CH	7.36, d (1.6)	7”/7”’, 10”/10”’, 11”/11”’, 13”/13”’
10” , 10”’	111.4, C		
11”,11”’	153.1, C		
12”, 12”’	112.7, C		
13”, 13”’	130.5, CH	7.16, d (1.6)	7’’/7’’’, 9’’/9’’’, 10’’/10’’’, 11’’/11’’’
NMR solvents used for **2** was CD_3OD at 600 MHz.			

Table S3. *Pseudomonas aeruginosa* strains used in the study

Name and Genotype	Description[a]	Reference
PAO1	Wild type; ATCC *Pseudomonas aeruginosa*	[1]
PAO1-*gfp*	GFP-tagged wild-type *Pseudomonas aeruginosa*	[2]
PAO1 *lasB-gfp*	Gm^r; PAO1 containing *lasB-gfp*(ASV) reporter fusion	[1]
PAO1 *rhlA-gfp*	Gm^r; PAO1, *rhlA-gfp*(ASV) reporter fusion	[3]
PAO1 Δ*lasI* Δ*rhlI*	Gm^r; PAO1 *lasI* and *rhlI* mutant	[4]

[a] Description of the strains' antibiotic resistance: Gm^r, gentamicin resistance.

References

[1]. Hentzer, M.; Riedel, K.; Rasmussen, T.B.; Heydorn, A.; Andersen, J.B.; Parsek, M.R.; Rice, S.A.; Eberl, L.; Molin, S.; Høiby, N.; Kjelleberg, S.; Givskov, M. Inhibition of quorum sensing in *Pseudomonas aeruginosa* biofilm bacteria by a halogenated furanone compound. *Microbiology* **2002**, *148*, 87–102.

[2]. Yang, L.; Barken, K.B.; Skindersoe. M.E.; Christensen, A.B.; Givskov, M.; Tolker-Nielsen, T. Effects of iron on DNA release and biofilm development by *Pseudomonas aeruginosa*. *Microbiology* **2007,** *153*, 1318–1328.

[3]. Yang, L.; Rybtke, M.T.; Jakobsen, T.H.; Hentzer, M.; Bjarnsholt, T.; Givskov, M.; Tolker-Nielsen, T. Computer-aided identification of recognized drugs as *Pseudomonas aeruginosa* quorum-sensing inhibitors. *Antimicrob. Agents Chemother.* **2009**, *53*, 2432–2443.

[4]. Hentzer, M.; Wu, H.; Andersen, J.B.; Riedel, K.; Rasmussen, T.B.; Bagge, N.; Kumar, N.; Schembri, M.A.; Song, Z.; Kristoffersen, P.; Manefield, M.; Costerton, J.W.; Molin, S.; Eberl, L.; Steinberg, P.; Kjelleberg, S.; Høiby, N.; Givskov, M. Attenuation of *Pseudomonas aeruginosa* virulence by quorum sensing inhibitors. *EMBO J.* **2003**, *22*, 3803–3815.
